# Classification of breast cancer histology images using Convolutional Neural Networks

**DOI:** 10.1371/journal.pone.0177544

**Published:** 2017-06-01

**Authors:** Teresa Araújo, Guilherme Aresta, Eduardo Castro, José Rouco, Paulo Aguiar, Catarina Eloy, António Polónia, Aurélio Campilho

**Affiliations:** 1 Faculdade de Engenharia da Universidade do Porto (FEUP), R. Dr. Roberto Frias s/n, 4200-465 Porto, Portugal; 2 Instituto de Engenharia de Sistemas e Computadores - Tecnologia e Ciência (INESC-TEC), R. Dr. Roberto Frias, 4200 Porto, Portugal; 3 Instituto de Investigação e Inovação em Saúde (i3S), Universidade do Porto, Rua Alfredo Allen, 208, 4200-135 Porto, Portugal; 4 Instituto de Engenharia Biomédica (INEB), Universidade do Porto, Rua Alfredo Allen, 208, 4200-135 Porto, Portugal; 5 Laboratório de Anatomia Patológica, Ipatimup Diagnósticos, Rua Júlio Amaral de Carvalho, 45, 4200-135 Porto, Portugal; 6 Faculdade de Medicina, Universidade do Porto, Alameda Prof Hernâni Monteiro, 4200-319 Porto, Portugal; Universita degli Studi di Torino, ITALY

## Abstract

Breast cancer is one of the main causes of cancer death worldwide. The diagnosis of biopsy tissue with hematoxylin and eosin stained images is non-trivial and specialists often disagree on the final diagnosis. Computer-aided Diagnosis systems contribute to reduce the cost and increase the efficiency of this process. Conventional classification approaches rely on feature extraction methods designed for a specific problem based on field-knowledge. To overcome the many difficulties of the feature-based approaches, deep learning methods are becoming important alternatives. A method for the classification of hematoxylin and eosin stained breast biopsy images using Convolutional Neural Networks (CNNs) is proposed. Images are classified in four classes, normal tissue, benign lesion, *in situ* carcinoma and invasive carcinoma, and in two classes, carcinoma and non-carcinoma. The architecture of the network is designed to retrieve information at different scales, including both nuclei and overall tissue organization. This design allows the extension of the proposed system to whole-slide histology images. The features extracted by the CNN are also used for training a Support Vector Machine classifier. Accuracies of 77.8% for four class and 83.3% for carcinoma/non-carcinoma are achieved. The sensitivity of our method for cancer cases is 95.6%.

## Introduction

Breast cancer is the first cause of death by cancer in women aged between 20 and 59 years and the second for women aged more than 59 years [1]. The diagnosis and treatment of this pathology in the early stages is essential to prevent the progression of the disease and reduce its morbidity rates [2].

**Fig 1:**
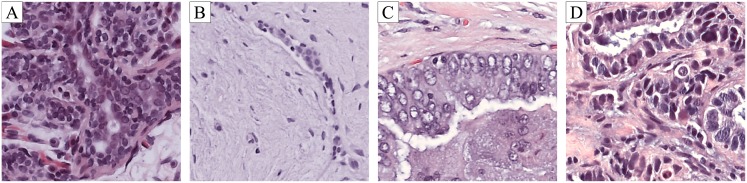
Examples of microscopy image patches from the used dataset [3]. Nuclei and cytoplasm appear purple and pinkish, respectively, due to the hematoxylin and eosin staining. **A** normal tissue; **B** benign abnormality; **C** malignant carcinoma *in situ*; **D** malignant invasive carcinoma.

Breast cancer diagnosis usually consists in an initial detection via palpation and regular check-ups using mammography or ultrasound imaging. The diagnosis is then followed by breast tissue biopsy if the check-up exam indicates the possibility of malignant tissue growth [4]. Breast tissue biopsies allow the pathologists to histologically assess the microscopic structure and elements of the tissue. The histology allows to distinguish between normal tissue, non-malignant (benign) and malignant lesions and to perform a prognostic evaluation [5]. Benign lesions represent changes in normal structures of breast parenchyma that are not directly related with progression to malignancy. Carcinomas can be classified as *in situ* or invasive. In *in situ* carcinoma the cells are restrained inside the mammary ductal-lobular system, whereas in invasive carcinoma the cells spread beyond that structure. The tissue collected during the biopsy is commonly stained with hematoxylin and eosin (H&E) prior to the visual analysis performed by the specialists. During this procedure, relevant regions of whole-slide tissue scans are assessed [6]. Fig 1 shows an example of patches from whole slide images stained with H&E for each of the classes mentioned. The staining enhances nuclei (purple) and cytoplasm (pinkish), as well as other structures of interest [7].

During the analysis of the stained tissue, pathologists analyze overall tissue architecture, along with nuclei organization, density and variability. For instance, tissues with invasive carcinoma show a distortion of the architecture as well as higher nuclei density and variability (Fig 1-D), whereas in normal tissue the architecture is maintained and the nuclei are well organized (Fig 1-A).

The diagnosis process using H&E stained biopsies is not trivial, and the average diagnostic concordance between specialists is approximately 75% [8]. The manual examination of histology images requires intense workload of highly specialized pathologists. The subjectivity of the application of morphological criteria in usual classification motivates the use of computer-aided diagnosis (CAD) systems to improve the diagnosis efficiency and increase the level of inter-observer agreement [9].

### Related work

CAD systems are embed Image Analysis and Machine Learning Methodologies developed to help physicians during the diagnosis procedure. Being a second opinion system, CAD systems reduce the workload of specialists, contributing to both diagnosis efficiency and cost reduction. For that purpose, there’s often an attempt to replicate the physicians’ method. For instance, the analysis of nuclei morphology may be sufficient to classify a tissue as benign or malignant [10].

Consequently, some works focus on nuclei analysis for malignant-benign classification. Kowal *et al.* [11] used different clustering algorithms for nuclei segmentation on fine needle biopsy microscopic images. Morphological, topological and texture features were used for training a classifier, achieving an accuracy between 84% and 93% on 500 images from 50 patients. Patient-wise classification was performed by majority voting on 10 images each, with an accuracy of 96–100%. Similarly, Filipczuk *et al.* [12] and George *et al.* [13] extracted nuclei-based features from fine needle biopsies. First, the circular Hough transform was used for detecting nuclei candidates, followed by false-positive reduction with machine-learning and Otsu thresholding. In George *et al.* [13] the nuclei segmentation is further refined using watershed. In both works, shape and texture features of nuclei are used for training different classifiers. Filipczuk *et al.* [12] achieved an accuracy of 98.51%, by majority voting over 11 images for each of the 67 patients, and George *et al.* [13] between 71.9% and 97.15% in individual image classification using 92 images. Besides nuclei-related information, Belsare *et al.* [14] also considered tissue organization for the binary classification of more complex images. The authors evaluated 70 images from a private 40× magnification breast histology *H*&*E* dataset. Spatio-color-texture graphs were used for segmenting the epithelial layer around the lumen of the cells, and statistical texture features were used for training the final classifiers. The authors report accuracies between 70% and 100%.

Other authors have focused on a more complex 3-classes classification of breast cancer histology images. For instance, both Brook *et al.* [15] and Zhang *et al.* [16] classified breast cancer tissue images in normal, *in situ* carcinoma and invasive carcinoma. For that, a dataset from the Israel Institute of Technology was used [17]. Brook *et al.* [15] binarized each image using multiple threshold values and used connected component statistics to train a support vector machine (SVM) classifier, reporting 93.4% average accuracy that could be increased to 96.4% by rejecting 20% of the images. Zhang *et al.* [16] used a cascade classification approach. Subsets of Curvelet Transform and local binary pattern (LBP) features were randomly fed to a first set of parallel SVM classifiers. Images where a given number of classifiers to disagree were rejected and analyzed by a second set of artificial neural networks (ANN) over other random feature subsets. Once again, images for which a certain number of classifiers disagreed were rejected. This system achieved 97% accuracy with 0.8% rejection rate.

The recent increase in available computing power and dataset sizes allowed the application of Convolutional Neural Networks (CNNs) to image classification problems. Contrarily to the traditional approach of hand-crafted feature extraction methods, CNNs learn useful features directly from the training image patches by the optimization of the classification loss function. These deep learning models have achieved excellent performance in image classification challenges in different fields [18, 19], including medical image analysis [20], and in particular on histopathology images [21].

CNNs allow to reduce the field-knowledge needed to design a classification system. Because of this, the performance of the methods is less biased by the dataset used and similar network architectures can achieve good results on different problems. In fact, Spanhol *et al.* [22] used a CNN architecture inspired in the Imagenet network [18] to classify *H*&*E* breast tissue biopsy samples in benign and malignant tumors, using multiple magnifications. In their work, 32 × 32 and 64 × 64 pixels patches were extracted from the initial images and used for training the CNN. The final classification was obtained by combining the patch probabilities with sum, product or maximum rules. Two patch extraction methods were studied, sliding window and random extraction. The extraction of patches allowed to reduce the complexity of the model by decreasing the size of the input in subsequent layers. The authors reported an accuracy decrease for higher magnifications, which suggests that their CNN architecture cannot extract relevant features for higher magnifications. In fact, for higher magnifications only nuclei edge-related features are extracted, as it will be discussed in the paper.

Other authors have adjusted the architecture of the CNN to breast histology related-problems with success. For instance, Ciresan *et al.* [19] used 101 × 101 patches to train a CNN for mitosis detection in H&E stained breast biopsy slides. The used architecture allows to study nuclei of different sizes and their neighborhoods. This methodology won the ICPR 2012 Mitosis Detection Contest with a F1-score of 0.782. In Cruz-Roa *et al.* [23] a CNN was trained on 100 × 100 pixels whole-slide patches, extracted using grid sampling, to detect invasive carcinoma regions in breast histology slides. Due to the global nature of the problem, their CNN feature-extraction scale ranges from nuclei to overall tissue organization. This method outperformed other state-of-the-art methods, achieving a F1-score of 0.780. For these last two works, the model was slided through the image to obtain a probability map and then the detection result was obtained via thresholding. In [19] the training dataset size and complexity were increased by applying arbitrary rotations and mirroring to the training instances.

### Contributions

In our work, a CNN designed for the analysis of breast cancer H&E stained histology images is proposed. Unlike previous approaches we perform image-wise classification in four classes of medical relevance: i) normal tissue, ii) benign lesion, iii) *in situ* carcinoma and iv) invasive carcinoma.

For this, a new breast cancer image dataset is presented. In addition, the proposed CNN architecture is designed to integrate information from multiple histological scales, including nuclei, nuclei organization and overall structure organization. By considering scale information, the CNN can also be used for patch-wise classification of whole-slide histology images. A data augmentation method is adopted to increase the number of cases in the training set. A SVM classification using the features extracted by the CNN is also used for comparison purposes.

## Materials and methods

### Dataset

The image dataset is composed of high-resolution (2040 × 1536 pixels), uncompressed, and annotated H&E stain images from the Bioimaging 2015 breast histology classification challenge [3]. All the images are digitized with the same acquisition conditions, with magnification of 200× and pixel size of 0.42*μm* × 0.42*μm*. Each image is labeled with one of four classes: i) normal tissue, ii) benign lesion, iii) *in situ* carcinoma and iv) invasive carcinoma The labeling was performed by two pathologists, who only provided a diagnostic from the image contents, without specifying the area of interest for the classification. Cases of disagreement between specialists were discarded. The goal of the challenge is to provide an automatic classification of each input image.

The dataset is composed of an extended training set of 249 images, and a separate test set of 20 images. In these datasets, the four classes are balanced. The images were selected so that the pathology classification can be objectively determined from the image contents. An additional test set of 16 images is provided with images of increased ambiguity, which we denote as “extended” dataset. The training and test datasets are publicly available at https://rdm.inesctec.pt/dataset/nis-2017-003.

### Preprocessing

**Fig 2:**
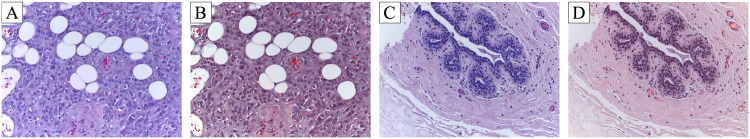
Histology image normalization. **A** and **C** original images; **B** and **D** images after normalization.

Prior to analysis, images are normalized using the method proposed in [24]. This method takes into account the staining technique used for the histology slides preparation. First, the colors of the images are converted to optical density (OD) using a logarithmic transformation. Then, singular value decomposition (SVD) is applied to the OD tuples to find the 2D projections with higher variance. The resulting color space transform is then applied to the original image. Finally, the image histogram is stretched so that the dynamic range covers the lower 90% of the data. Fig 2 shows two images before and after normalization.

### Image-wise classification

In the work herein described, image classification is performed by first processing several patches with a patch-wise classifier, and then combining the classification results of all the image patches to obtain the final image-wise classification.

The classification of breast cancer histology images into one of the four target classes must rely on the extraction of nuclei related features as well as features related to overall tissue organization. The nuclei features are useful to differentiate between carcinoma and non-carcinoma cells, and should include single nucleus information, such as color and shape, as well as nuclei organization features like density or variability. Differently, tissue structure information is necessary to differentiate between *in situ* and invasive carcinomas. Thus, the classification should be based on features which scales range from less than the size of a nucleus to several nuclei wide.

The visual analysis of the dataset images indicates that nuclei radius ranges from 3 to 11 pixels (1.26*μm* to 4.62*μm*). Also, in our initial observations we postulated that patches of about 128 × 128 pixels should be enough to cover the relevant tissue structures. However, in our dataset the label is assigned to the whole image of 2040 × 1536 pixels, meaning that there is no guarantee that small regions contain relevant diagnosis information. This motivated the use of larger image patches of 512 × 512 pixels to ensure that a more reliable label can be provided for each image patch. A patch dataset is generated from the training dataset as explained in section *Augmented patch dataset*.

The procedure to classify one image is as follows. First the original image is divided into twelve contiguous non-overlapping patches. The patch class probability is computed using the patch-wise trained CNN and CNN+SVM classifiers. Then, the image-wise classification is obtained using one of three different patch probability fusion methods: i) majority voting, where the image label is selected as the most common patch label, ii) maximum probability, where the patch with higher class probability decides the image label and iii) sum of probabilities, where the patch class probabilities are summed and the class with the largest value is assigned. Draws are solved by prioritizing malignant classes using the following order: i) invasive, ii) *in situ*, iii) benign and iv) normal. This criterion increases the sensitivity of our approach for the carcinoma classes in detriment of the non-carcinoma classes, which is of greater interest for a second-opinion system.

### Augmented patch dataset

An augmented patch dataset is created from the normalized images in the training set. The used dataset has a low number of samples when compared to other CNN classification problems [18]. The network might thus be prone to overfit. Dividing images into patches allows to increase the dataset complexity and dimension. Data augmentation through patch rotation and mirroring further improves the dataset. This is possible because the studied problem is rotation invariant, i.e., physicians can study breast cancer histological images from different orientations without altering the diagnosis. Consequently, rotations and mirroring allow to increase the size of the dataset without deteriorating its quality. Patching and dataset augmentation have already been used successfully on similar histological classification problems [19]. However, they have not been used for carcinoma classification.

First, the image is divided in patches of 512 × 512 pixels size, with 50% overlap. Some example patches are shown in Fig 1. Patch normalization is performed by subtracting the average value to the red, green and blue channels separately. Each patch is then transformed into eight different patches by combining *k* ⋅ *π*/2 rotations, with *k* = {0, 1, 2, 3}, and vertical reflections. This results in a total of 70000 different patches from the original 250 training images. Each of the patches is considered to have the same class label as the original image.

### CNNs for patch-wise classification

CNNs are used for classifying the 512 × 512 histology image patches into the four tissue classes. CNNs are feed-forward neural networks that are specialized in visual pattern recognition. Neurons are connected to overlapping local image patches (receptive fields), and arranged in convolutional maps with all the neurons sharing the same weights. This allows the convolutional maps to act as local image filters, detecting the same patterns at all the image positions, and to reduce the total number of parameters to be trained [25]. The network is organized in a hierarchical layer structure that, at each level, combines the lower level features into higher level ones, until the image class label is obtained.

**Fig 3:**
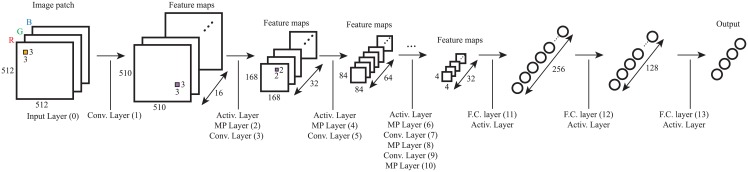
Convolutional Neural Network architecture, according to Table 1. The original image has 512 × 512 pixels and 3 RGB channels. Orange and purple squares represent the convolutional and max-pooling kernels, respectively.

The proposed network architecture follows the common trends in previous successful applications of CNNs for image classification [18, 19, 26], with several convolutional-pooling layer pairs, followed by a fully-connected network. The architecture providing the best results in our experiments is summarized in Table 1, and illustrated in Fig 3, and resulted from the following design considerations:

- Input layer: The input layer has three channels of 512 × 512 pixels, corresponding to the normalized RGB patches extracted from the images.
- Depth and number of maps: As previously discussed, breast cancer tissue classification requires the analysis at several feature scales. In the target images the nuclei radii are between 3 and 11 pixels, and it is required to explore nuclei-scale features, nuclei organization features, and structure-scale features. The proposed network architecture has, therefore, convolutional layers with enough neural maps to represent each of these three features at their range of scales, as shown in Table 1. The final fully-connected network performs the integration of the information for the whole image patch, and provides the final classification. The large input size and multi-scale network design enable the extension of the method for whole-slide images.
- Max-pooling: The lower level information needs to be spatially integrated for the image region, as well as simplified when accounting for higher level information. Max-pooling layers allow for such a complexity reduction without increasing the number of parameters in the network. The max pooling layers use a stride equal to the pooling size;
- Non-saturating nonlinearity: Both the convolutional layers and fully-connected layers are composed of Rectified Linear Units, with activation function *f*(*x*) = max(0, *x*) [27]. This non-linearity is selected to help avoiding the vanishing gradients and to improve the training speed [18, 27].
- Output layer: The output is composed of four neurons, corresponding to each of the four classes, that are normalized with a *softmax* activation function.

**Table 1:** Proposed Convolutional Neural Network architecture. Left side notes show the histological association with the network layers: **A**—edges; **B**—nuclei; **C**—nuclei organization; **D**—structure and tissue organization.

			Layer type	Maps & Neurons	Filter size	Effective receptive field	Effective receptive field (*μm*)
		0	Input	3M × 512 × 512N		1 × 1	0.4 × 0.4
	**A**	1	Convolutional	16M × 510 × 510N	3 × 3	3 × 3	1 × 1
		2	Max-pooling	16M × 170 × 170N	3 × 3	5 × 5	2 × 2
**C**	**B**	3	Convolutional	32M × 168 × 168N	3 × 3	11 × 11	4.6 × 4.6
		4	Max-pooling	32M × 84 × 84N	2 × 2	14 × 14	5.9 × 5.9
		5	Convolutional	64M × 84 × 84N	3 × 3	26 × 26	11 × 11
		6	Max-pooling	64M × 42 × 42N	2 × 2	32 × 32	13 × 13
	**D**	7	Convolutional	64M × 42 × 42N	3 × 3	56 × 56	24 × 24
		8	Max-pooling	64M × 14 × 14N	3 × 3	80 × 80	34 × 34
		9	Convolutional	32M × 12 × 12N	3 × 3	152 × 152	63.8 × 63.8
		10	Max-pooling	32M × 12 × 12N	3 × 3	224 × 224	94.1 × 94.1
		11	Fully-connected	256N		512 × 512	215 × 215
		12	Fully-connected	128N		512 × 512	215 × 215
		13	Fully-connected	4N		512 × 512	215 × 215

The model is trained with 75% of the training set, and validated on the remaining images. The validation set is randomly selected for each epoch. The training process stops after the stabilization of the validation accuracy with equal weight for all the classes (50 epochs). The network weights are initialized randomly, and an adaptive learning rate gradient-descent back-propagation algorithm is used for weight update [28]. The selected loss function is categorical cross entropy.

For comparison, the features extracted by the CNN are used for training a Support Vector Machine classifier (CNN+SVM). The activations of the second fully connected layer are used as features. A radial basis function kernel is used and the optimal parameters are obtained by exhaustive search using 3-fold cross validation on the training data. The classifier is trained using the whole training set.

### Results evaluation

The performance of our method is evaluated in terms of sensitivity and accuracy. This evaluation is performed patch-wise and image-wise for the initial and extended sets. A binary classification in non-carcinoma and carcinoma is also considered by grouping normal with benign outcomes and *in situ* with invasive results, respectively. Table 2 details the number of images and patches used.

**Table 2:** Number of images (and patches) used for performance evaluation. A total of 36 images and 512 patches are considered.

Dataset	non-carcinoma		carcinoma	
	Normal	Benign	*in situ*	Invasive
**Initial**	10 (120)		10 (120)	
	5 (60)	5 (60)	5 (60)	5 (60)
**Extended**	8 (96)		8 (96)	
	4 (48)	4 (48)	4 (48)	4 (48)
**Overall**	18 (216)		18 (216)	
	9 (108)	9 (108)	9 (108)	9 (108)

## Results

### Patch-wise classification

The patch-wise accuracy and sensitivity are shown in Tables 3 and 4, respectively. The overall accuracy (initial plus extended datasets) is 66.7% for the CNN and 65.0% for the CNN+SVM classifier. The performance of our system is lower for the extended dataset due to its increased complexity. The overall accuracy increases when only two classes (non-carcinoma and carcinoma) are considered (77.6% for the CNN and 76.9% for the CNN+SVM). This indicates that the normal/benign and *in situ*/invasive classes share similar features between them. Furthermore, the proposed system achieves an overall sensitivity of approximately 81% for carcinoma patch-wise classification.

**Table 3:** Patch-wise accuracy (%) (2 and 4 classes).

Classifier	No classes	Initial	Extended	Overall
**CNN**	4	72.5	59.4	66.7
	2	80.4	74.0	77.6
**CNN+SVM**	4	72.9	55.2	65.0
	2	82.9	69.3	76.9

**Table 4:** Patch-wise sensitivity (%) (2 and 4 classes).

Dataset	Classifier	non-carcinoma		carcinoma	
		Normal	Benign	*in situ*	Invasive
**Initial**	**CNN**	69.2		91.7	
		61.7	56.7	83.3	88.3
	**CNN+SVM**	76.7		89.2	
		65.0	61.7	76.7	88.3
**Extended**	**CNN**	81.3		66.7	
		50	72.9	58.3	56.3
	**CNN+SVM**	82.3		56.3	
		54.2	66.7	43.8	56.3
**Overall**	**CNN**	74.5		80.6	
		56.4	63.9	72.2	74.1
	**CNN+SVM**	79.2		74.5	
		60.2	63.9	62.0	74.1

### Image-wise classification

**Fig 4:**
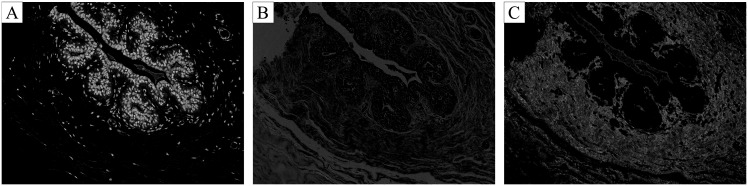
Activation examples for the first (A, B) and second (C) layers of the Convolutional Neural Network. Different structures with diagnostic relevance are analyzed.

Image-wise classification results are shown in Tables 5 and 6, respectively. Majority voting shows the best results, achieving an overall accuracy of 77.8% for four classes. These results are constant regardless of using CNN or CNN+SVM for patch-wise classification. In both methods maximum probability is the worst performing method suggesting it is not a suitable strategy for this problem. Regarding binary classification, the overall accuracy increases for both classifiers when compared to the four class problem. Furthermore, CNN+SVM seems to outperform the CNN model, achieving a total accuracy of 83.3% for the best voting methods. In comparison, CNN’s performance is only better for the extended set using majority voting. The lower accuracy of patch-wise classification is explained by the fact that patch labels are obtained from the image labels without any information about the location of the abnormalities. This approach is sub-optimal as, regardless of the image class, normal tissue regions may also be present. As a result, noise is introduced in the training set, contributing to the lower patch-wise accuracy. Despite this, the network is focusing relevant details of the image. For instance, Fig 4 shows activations of first and second layers of the CNN, where relevant diagnosis structures, such as nuclei or stroma organization of low and high nuclei density regions, are being prioritized.

**Table 5:** Image-wise accuracy (%) using different voting rules (2 and 4 classes).

Classif.	Vote	4 Classes			2 Classes		
		Init.	Exten.	Overall	Init.	Exten.	Overall
**CNN**	Maj.	80.0	75.0	77.8	80.0	81.3	80.6
	Max.	80.0	62.5	72.2	80.0	75.0	77.8
	Sum	80.0	68.8	75.0	80.0	75.0	77.8
**CNN+SVM**	Maj.	85.0	68.8	77.8	90.0	75.0	83.3
	Max.	80.0	62.5	72.2	80.0	75.0	77.8
	Sum	85.0	68.8	77.8	90.0	75.0	83.3

**Table 6:** Image-wise sensitivity (%) using majority voting (2 and 4 classes).

Dataset	Classifier	non-carcinoma		carcinoma	
		Normal	Benign	*in situ*	Invasive
**Initial**	**CNN**	70		90	
		80	40	100	100
	**CNN+SVM**	80		100	
		80	60	100	100
**Extended**	**CNN**	50		100	
		75	75	75	75
	**CNN+SVM**	50		90	
		75	75	50	75
**Overall**	**CNN**	61.1		94.4	
		77.8	55.6	88.9	88.9
	**CNN+SVM**	66.7		95.6	
		77.8	66.7	77.8	88.9

### Feature visualisation

**Fig 5:**
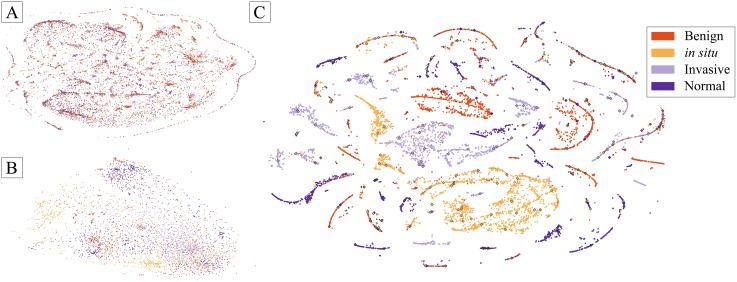
2D projection of the training patches and their activations on different layers of the CNN using t-SNE [29]. **A** training patches; **B** last convolutional layer; **C** second fully-connected layer. Diamond shapes represent test images.

Fig 5 shows a two-dimensional representation of the initial training set and the activations of the last convolutional and the second fully-connected layers. These representations result from the application of t-SNE, which is an efficient parametric embedding technique for dimensionality reduction that preserves distance between samples [29]. In these representations, each point corresponds to a patch and the 2D distance between points is an approximation of the original Euclidean distance in the multidimensional space. In Fig 5-C, test set patches are also represented. As shown in Fig 5-A and 5-B, the CNN tends to approximate samples of the same class in higher layers. This indicates these layers are extracting relevant features from the initial data after training. In Fig 5-C, patches appear organized in clusters dominated by one class, indicating a good differentiation between patches with different labels after the two fully-connected layers. Differently, the presence of points of different classes possibly represents misclassified patches. Despite this, the overall patch organization indicates that the the fully-connected layer activations are useful features for classification using the suggested SVM model.

### Comparison with the state-of-the-art

CNNs were used by Cruz-Roa *et al.* [23] to perform classification of whole slide high-resolution image patches as invasive carcinoma. The achieved sensitivity was 79.6%. The overall sensitivity of our method for patch-wise classification of invasive carcinoma is 74.1%. These results are not directly comparable due to several reasons: 1. our method discriminates patches in 4 classes as opposed to the segmentation problem considered in [23], which focuses only on invasive carcinoma and non-invasive carcinoma region classification; 2. in the previous work, patch-wise whole-slide images ground truth is available. In our case, only image-wise ground truth corresponding to a smaller section in the whole-slide image is available. Thus, in our dataset some patches in the training and testing sets may not contain relevant information to be correctly classified, lowering the accuracy in patch classification.

Despite this, our method performance shows to be close to that of [23], specially when considering that ours is not a dedicated invasive carcinoma detection method. For the CNN architecture and image resolution in [23], spatial related features with size between 4*μm* and 100*μm* are analyzed by their algorithm. The diameter of breast cells nuclei is approximately 6*μm*, which suggests that sub-nuclei features such as texture are not being considered. This indicates that the good classification results reported by the authors are based on tissue organization features. By comparison, our architecture is able to capture features with size between 1.3*μm* and 94*μm*. This allows the CNN to learn not only individual nuclei features but also the structures’ organization.

In the work of Spanhol *et al.* [22] CNNs were used for classifying breast cancer histology images of different magnifications in benign or malign tumours. For the 200× magnification, the achieved accuracy was approximately 84%. In our work, the overall image-wise accuracy for the non-carcinoma/carcinoma tissue classification is approximately 81% when using CNN and 83% with SVM classifier. The methods present similar performances, even though our training was performed considering 4 classes. Besides, the dataset used in [22] contains approximately 2000 images for the referred magnification, which is a significantly larger training set. We were able to train a more complex model with less training examples thanks to the proposed data augmentation method. Further, in [22] images were selected in such a way that only relevant regions for diagnosis were present, while in our case non-relevant regions for classification were present both in the patch-wise training and testing set which can mislead the network training.

Considering the CNN architecture and image resolution in Spanhol *et al.* [22] spatial related features with size between 0.2*μm* and 7*μm* are learned for the 200× magnifications. However, if the nuclei diameter is approximately 6*μm*, then the reported network architecture is not able to learn features at higher scales through its convolutional layers. Furthermore, the authors use the same CNN architecture for different amplifications, implying that larger features are learned for lower magnifications. As discussed previously, nuclei organization is also relevant for the diagnosing process. They achieve better results for lower magnifications, indicating that taking care of the relevant scale analysis is important for having success with CNN architectures for classification. By comparison our more complex architecture is suitable for learning features at multiple relevant scales.

## Conclusions

A CNN-based approach for the classification of H&E stained histological breast cancer images is proposed. All relevant features are learned by the network, reducing the need of field knowledge. Images are classified as either normal tissue, benign lesion, *in situ* carcinoma and invasive carcinoma. Alternatively, a binary classification as carcinoma or non-carcinoma is also performed. For this, the architecture of the network is designed to extract information from different relevant scales, including nuclei and overall tissue organization. The network is trained on an augmented patch dataset and tested on a separate set of images. Both dataset augmentation and scale-based network design have been shown important for the success of the approach. The extracted features are also used for training a SVM classifier. Both CNN and SVM classifiers achieve comparable results. The proposed classification scheme allows to obtain high sensitivity for carcinoma cases, which is of interest for pathologists. The performance of our system is similar or superior to the state-of-the-art methods, even though a smaller and more challenging dataset is used. Finally, since the network is designed to consider multiple biological scales, the proposed system can be extended for whole-slide breast histology image classification relevant for clinical settings.
